# Cross-species multi-omic analyses of great ape ejaculates reveal novel strategies for enhancing sperm cryopreservation in Bornean orangutan (Pongo pygmaeus)

**DOI:** 10.1186/s40851-026-00262-x

**Published:** 2026-03-06

**Authors:** Laura Orama Méar, Yu-Chia Chang, Jane-Fang Yu, Yun-Chen Hsieh, Chia-Lin Hsu, Cheng-Chih Hsu, Pei-Shiue Tsai

**Affiliations:** 1 Graduate Institute of Veterinary Medicine, National Taiwan University, Taipei, Taiwan; 2 Department of Reproduction Biology, Leibniz Institute for Zoo & Wildlife Research, Berlin, Germany; 3 Conservation and Research Center, Taipei Zoo, Taipei, Taiwan; 4 Department of Chemistry, National Taiwan University, Taipei, Taiwan; 5 Department of Veterinary Medicine, National Taiwan University, Taipei, Taiwan; 6 Leeuwenhoek Laboratories Co. Ltd, Taipei, Taiwan; 7 Research Center for Developmental Biology and Regenerative Medicine, National Taiwan University, Taipei, Taiwan

**Keywords:** Orangutan, Sperm, Cryopreservation, Proteomic, Lipidomic

## Abstract

The orangutan (*Pongo pygmaeus* [Bornean] and *Pongo abelii* [Sumatran], Linnaeus, 1760) is the most endangered of the great apes, classified as Critically Endangered. Sperm cryopreservation is a valuable tool for banking genetic resources and solving the complexities of relocating animals; however, cryopreservation protocols remain suboptimal for this species. Due to their phylogenetic closeness, this study aimed to explore the applicability of a chimpanzee (*Pan troglodytes*, Blumenbach, 1775) sperm cryopreservation protocol to orangutans. To guide further modifications to the protocol, we revealed, for the first time, the comprehensive lipidomic and proteomic characterizations of orangutan ejaculates with parallel comparisons to chimpanzee ejaculates. Functional analyses of oxidative and osmotic stress responses were also conducted to provide valuable evidence of the physiological changes and defense mechanisms associated with sperm cryodamage. The cross-species multi-omic analyses revealed that, compared to the chimpanzee, the orangutan sperm lipid profile exhibited significant alterations after the freezing-thawing process, notably characterized by a substantial loss of cholesterol. While interspecies differences in antioxidant enzyme composition and activity were observed, there was insufficient evidence to support a heightened susceptibility of orangutans to oxidative stress. Conversely, orangutan sperm exhibited low tolerance to hypoosmotic conditions. To prevent cryodamage, a modified thawing protocol that implemented a serial dilution approach significantly minimized hypoosmotic shock and improved post-thaw motility to 19%. In conclusion, this study presents the first complete proteome and lipidome analyses of chimpanzee and orangutan ejaculates, providing valuable insights into the physiological changes and defense mechanisms associated with sperm cryopreservation. This knowledge enabled a science-based approach to improving cryopreservation protocols, moving beyond empirical trial-and-error.

**Supplementary Information:**

The online version contains supplementary material available at 10.1186/s40851-026-00262-x.

## Background

 Species extinction has occurred since the beginning of life on Earth [1]. According to the IUCN Red List, a cycle of extinction is already underway, following an almost domino effect, as nearly one in four animals is threatened with extinction. These numbers are even more alarming within the Hominidae family, the great apes, where all species are either endangered or critically endangered with extinction, except for humans [2]. The orangutan (*Pongo spp*., Linnaeus, 1760) is the most affected genus within the Hominidae family [3]. Many factors including habitat loss, overhunting, and low reproductive capacity, have been hypothesized to affect orangutan populations [4]. Despite orangutan rehabilitation and reintroduction camps implemented over the past few decades, high mortality, especially among young orangutans, hampers reintroduction success [5]. Unfortunately, as the threats against the wild population continue to increase, in situ conservation efforts do not seem to withstand a chance to fully protect endangered animals from extinction. Therefore, efforts to preserve populations outside of their habitat via *ex situ* conservation strategies become an imminent need [6, 7]. However, genetic diversity in fragmented and small populations has proven to be a challenge as the animals are often genetically closely related, subspecific hybrids, and sensitive to transport [6, 8]. Improvement of genetic diversity in captive populations can be obtained through reproductive management of the species [9].

Sperm cryopreservation is a valuable tool to address these challenges, as the frozen genetic material can be directly transported to another location for the application of artificial reproductive techniques to obtain genetically diverse offspring [6]. A recent study by Zainuddin and colleagues in 2022 reported a pilot study on orangutan sperm cryopreservation using a 6% glycerol and egg yolk buffer. Although they showed post-thaw sperm motility of 5.00 ± 1.87% [7], the outcome was likely impacted by several forms of cryodamage, including ice crystal formation, osmotic stress, oxidative stress, and alterations in membrane lipid content [10–12]. Sharp ice crystals damage the sperm membranes [13] and cause hypertonic stress [14]. During thawing, the reverse process takes place as water becomes liquid again and sperm cells are exposed to sudden hypotonic conditions [11]. Furthermore, loss of antioxidant enzymes and reactive oxygen species scavengers is also observed [15]. The combination of lipid phase transition, peroxidative damage, and significant loss of cholesterol and phospholipids renders the sperm membrane more prone to damage and breakage [12, 16].

Our earlier study established a successful cryopreservation protocol in chimpanzees (*Pan troglodytes*, Blumenbach, 1775), which demonstrated post-thaw sperm motility of 50% [17, 18]. Due to the phylogenetic closeness between chimpanzees and orangutans, we hypothesize that applying the cryopreservation protocol used in chimpanzees will improve the earlier reported post-thaw sperm quality in orangutans. Moreover, we strongly believe that revealing the species-specific cryodamages that occur during cryopreservation, through comprehensive proteomic and lipidomic comparisons, will also provide vital information necessary to develop a more effective cryopreservation protocol for orangutans. Thus, the aim of the present study is to apply multi-omic approaches for systematic comparisons between chimpanzee and orangutan ejaculates to optimize orangutan sperm cryopreservation.

## Materials and methods

### Study design

The present work was an experimental study structured into three phases: Phase 1 involved the application of a well-established chimpanzee sperm cryopreservation protocol to orangutan sperm, followed by post-thaw evaluation of sperm quality. Phase 2 comprised a cross-species comparison of the lipidomic and proteomic profiles of ejaculates with the aim of elucidating responses and defense mechanisms against cryodamage. Phase 3 involved optimization of the cryopreservation protocol based on evidence obtained from earlier phases. The experimental unit consisted of an individual ejaculate collected from captive adult male orangutans (total number of ejaculations = 10) or chimpanzees (total number of ejaculations = 15) throughout the year. In Phase 3, each ejaculate was subjected to all treatments without randomization. The sample size was determined by logistical constraints to animal availability. No blocking system was implemented. No a priori power analysis was performed. The population of inference consists of captive orangutans and chimpanzees housed in zoological institutions.

### Chemicals and reagents

Chemicals and reagents were obtained from Sigma Aldrich (Missouri, USA) unless stated otherwise. Sperm incubation medium Ham’s F-12 nutrient mix (11765-054), later referred to as F-12, was purchased from Thermo Fisher Scientific Inc. (Oregon, USA). Monoclonal antibodies against aquaporin 5 (SC-514022), aquaporin 7 (SC-376407), and polyclonal antibody against aquaporin 11 (aqp11-1101sp) were acquired from Santa Cruz Biotechnology (California, USA) and FabGennic International Inc. (Texas, USA), respectively. All immunofluorescence secondary antibodies were obtained from Jackson Immuno-Research (Pennsylvania, USA).

### Animals, semen collection, and pre-analysis handling

Semen samples were collected from four adult chimpanzees and two adult Bornean orangutans (*Pongo pygmaeus*, Linnaeus, 1760) housed at Taipei Zoo. The chimpanzee colony consisted of males aged 7 to 23 years old, and the group was housed with a fixed feeding schedule and water available *ad libitum*. The Bornean orangutan colony was composed of a mixed group containing males, females, and an infant with a fixed feeding schedule and water *ad libitum*. The males’ ages ranged from 32 to 40 years old. Semen collection was carried out at Taipei Zoo in accordance with the regulations and approval of the animal welfare committee of Taipei Zoo (IACUC #11201). The chimpanzee semen collection was performed using an artificial vagina (IMV Ram/goat vagina 13.5, IMV Technologies, L’Aigle, France) (Supplementary Figure S1A, B). The orangutan semen collection was performed using hand massage (masturbation) due to the smaller size and shape of their penis (Supplementary Figure S1C, D). Ejaculates were collected into a 50 mL Falcon tube, and 1 mL of F-12 was added immediately to facilitate the liquefaction of the sperm plug. We demonstrated previously that adding F-12 medium to the sperm plug and incubating the mixture at 27˚C for 30 min enabled the majority of the sperm plug to liquefy [17]. The undissolved semen colloids and cell debris were further strain-separated through a 40 nm pore-size cell strainer.

### Semen cryopreservation and post-thaw analysis

The chimpanzee semen cryopreservation was carried out as previously described [17, 18]. Briefly, after liquefaction, the sperm suspension was placed in a water bath at 4˚C for 30 min. Egg yolk-free medium SpermCryo ™ All-round (SPCA-005, Gynotec B.V, The Netherlands), was used at a 1:3 dilution ratio to semen suspension in the presence of a final glycerol concentration of 6.6%. The mixture was kept at 4˚C for 10 min before being aliquoted into cryopreservation straws (0.25 mL) in a 4˚C chamber. The straws were subsequently exposed to nitrogen vapor (4 cm above the liquid nitrogen surface) for 10 min, then immersed and stored in liquid nitrogen at -196˚C. For post-thaw analyses, the cryopreserved straws were thawed in a 37 °C sterile water bath for 1 min. The contents were further diluted with 1 ml of pre-warmed F-12 and subjected to centrifugation at 500 × g for 7 min at 37 °C (Thawing protocol 1.0). For the optimized thawing protocol (Thawing Protocol 2.0), straws were thawed in a water bath at 37˚C for 10 s, followed by the slow addition (100 µl/min) of F-12 and processing at 21˚C. Following centrifugation, the supernatant was carefully aspirated to avoid disturbing the pellet. The pelleted sample was then resuspended in 200 µL of pre-warmed F-12. Post-thaw sperm motility was evaluated by computer-assisted sperm analysis (CASA, Hamilton Thorne Inc., Beverly, USA) or iSperm^®^, a portable sperm analysis device (Aidmics Biotechnology Co., LTD, Taiwan). The software and parameter settings were based on recommendations of Hamilton Thorne Inc., and image capture was set to 60 frames per second; a total of 45 frames were recorded per examination field. The default parameters and software were set up using human sperm parameters as a reference, with adjustments specifically for chimpanzee and orangutan use. At least 5 independent repeats were performed for each experimental condition; the mean value and standard deviation (SD) were calculated accordingly. Motility-related parameters, including total motility (%) and progressive motility (%, Hamilton system defined as VAP ≥ 25 μm/s, STR ≥ 30%), were measured and analyzed. Morphology was also evaluated according to the World Health Organization (WHO) classification [19].

### Lipidomic and proteomic analyses

The cryopreservation process alters enzyme activity and membrane lipid composition/organization, which further compromises post-thaw sperm quality [20, 21]. To allow comprehensive comparisons, proteomic and lipidomic analyses were conducted on both chimpanzee and orangutan ejaculates. Detailed methodology and sample processing workflow are described in the Supplementary Materials (S1 section).

For the lipidomic analysis (UPLC-MS/MS), lipid extraction from sperm samples was performed using a modified MTBE protocol with deuterated internal standards, followed by UPLC–Orbitrap Elite analysis using a CSH C18 column and positive-mode HESI (for untargeted metabolomics analysis) or APCI (for targeted cholesterol analysis). Data were aligned, normalized (SERRF), and annotated with Compound Discoverer and LipidSearch, with QC samples injected throughout the run. Statistical analysis (FDR < 0.05; FC > 2), PCA, and figure generation were conducted using MetaboAnalyst and GraphPad Prism. For proteomic analysis, seminal plasma and sperm cells were separated through centrifugation. A BCA assay was performed prior to SDS-PAGE and Coomassie staining. Gel slices were subjected to reduction, alkylation, and Lys-C/trypsin digestion. Peptides were analyzed by LC–MS/MS using an Orbitrap Fusion system, and the resulting spectra were searched against the SwissProt human database with Mascot. Identified proteins were converted to gene IDs and analyzed for functional enrichment and protein–protein interactions using DAVID and STRING. Related figures were created using STRING, R, and Cytoscape.

### Antioxidant enzymes evaluations

To specify the presence and tissue distribution of antioxidants, ejaculates were centrifuged at 1600 × g for 10 min at 4˚C to separate the seminal plasma from sperm cells. The supernatant (seminal plasma diluted in F-12) was separated from the sperm pellet, and both samples were flash-frozen in liquid nitrogen and then stored at -80˚C until further analysis. To determine the overall antioxidant capacity of chimpanzee and orangutan ejaculates, the Total Antioxidant Capacity Assay Colorimetric Kit (TAC) (ab65329, Abcam, United Kingdom) was used according to the manufacturer’s instructions. The total antioxidant capacity was calculated after 90 min of incubation and expressed in mM of Trolox. To study the antioxidant defense mechanisms of the ejaculates in detail, antioxidant enzyme assays were performed. Superoxide dismutase (SOD) activity, glutathione peroxidase (GPX) activity, and glutathione S-Transferase (GST) activity were assessed using Superoxide Dismutase Assay Kit (706002, Cayman Chemical, USA), Glutathione Peroxidase Assay Kit (703102, Cayman Chemical, USA), and Glutathione S-Transferase Activity Colorimetric Assay Kit (ab65326, Abcam, United Kingdom), respectively according to the manufacturer’s instructions.

### Osmotic stress evaluation

The osmotic tolerance of chimpanzee and orangutan spermatozoa, using motility as the endpoint, was determined by exposing sperm cells to F-12 medium of different osmolarities (150, 200, 250, 300, 350, 400, and 450 mOsm) and measuring motility after returning the sperm cells to isosmotic conditions (F-12; 270mOsm). The anisosmotic solutions were prepared using commercially available F-12 medium as a base medium (270 mOsm). The anisosmotic F-12 solutions lower than 270 mOsm (i.e., 150, 200, and 250mOsm) were prepared by diluting F-12 medium with DDW (pH 7-7.4). The anisosmotic F-12 solutions with osmolarities higher than 300 mOsm (i.e., 300, 350, 400, and 450 mOsm) were prepared by adding D-glucose to the F-12 medium to achieve the desired osmolarity. The osmolarity of solutions was determined using a freezing-point depression osmometer (Osmomat 3000 basic, Gonotec, Germany). After liquefaction, a 10 µL fixed concentration (450 × 10^6^ spermatozoa/ml) sperm aliquot was transferred into an Eppendorf containing 150 µL of the isosmotic (300 mOsm) or anisosmotic F-12 solutions (i.e., 150, 200, 250, 350, 400, and 450 mOsm). After incubation for 5 min at 25˚C, spermatozoa were returned to near isosmolarity (270–328mOsm) by mixing 100 µL anisosmotic sample with 200 µL F-12 medium solution. The total (TM) and progressive (PM) motility were then normalized using the following equation:

$$\eqalign { & Normalized\,motility\left({TM\,or\,PM} \right)\left(\% \right) \cr & = {{Motility\left({TM\,or\,PM} \right) \times 100} \over {Motility\left({TM\,or\,PM} \right)at\,270mOsm}} \cr}$$

Each post-handling value was compared to the value obtained in isosmotic conditions (270 mOsm, the osmolarity of the medium routinely used).

### Aquaporin distribution via indirect immunofluorescent staining

Aquaporins are the primary water channels responsible for volume regulation and play a crucial role in response to osmotic stress [22]. The cellular location of the different aquaporin isoforms in chimpanzee and orangutan spermatozoa was determined by indirect immunofluorescence staining. For every staining, negative controls consisting of staining without the primary antibody (replaced with 0.1% or 10% BSA) were performed. All samples were evaluated under the same conditions of exposure time and laser power using an IX83 inverted fluorescence microscope (Olympus, Japan).

To identify the presence and the cellular localization of the above-mentioned aquaporins, immunofluorescent staining of aquaporin 5 (mouse monoclonal, SC-514022) and aquaporin 7 (mouse monoclonal, SC-376407) was carried out according to the manufacturer’s instructions with slight modifications. Briefly, sperm cells were fixed with 4% paraformaldehyde (PFA) for 15 min at room temperature (RT). Permeabilization of fixed sperm was performed using 0.01% Triton X-100 for 3 min at RT. The non-specific signal was reduced using 10% BSA for 30 min at room temperature. Anti-aquaporin 5 and 7 at a dilution of 1/50 in 1% BSA were used for 1 h incubation at RT. Subsequently, Alexa Fluor 564 anti-mouse secondary antibody (dilution 1/150) was used for further incubation for 1 h at RT. The aquaporin 11 location was accessed following the primary antibody manufacturer’s instructions (rabbit monoclonal, aqp11-1101sp), with slight modifications. Briefly, sperm cells were fixed with 4% PFA for 15 min at RT. Permeabilization of fixed sperm was performed using 0.1% Triton X-100 for 15 min at RT. The non-specific signal was minimized by 2% BSA for 1 h incubation at RT. Aquaporin 11 was labeled using rabbit anti-aquaporin 11 at a 1/150 dilution in 0.1% BSA. The incubation was executed at RT for 3 h. Subsequently, the secondary antibody Alexa Fluor 564 anti-rabbit (1/150 dilution) was incubated for 1 h at RT.

### Statistical analysis

Statistical analysis was carried out using GraphPad Prism (version 8.0.1; Boston, USA). Results were expressed as the mean ± standard deviation (SD). All data were subjected to a Shapiro-Wilk test to determine if they presented a Gaussian distribution. Equal variances were also checked using the Bartlett test for homogeneity of variances. If the data presented an asymmetric distribution for any parameter, log transformation prior to analysis was used to obtain a normal distribution. If, after log transformation, the data still did not present a normal distribution, non-parametric statistical tests were used. Inter-species comparisons were analyzed by unpaired T-test or ANOVA (chimpanzee ejaculate vs. orangutan ejaculate/ chimpanzee sperm vs. orangutan sperm/ chimpanzee seminal plasma vs. orangutan seminal plasma). Data that were not normally distributed were analyzed by the Mann-Whitney comparison of ranks. Data presenting different standard deviations between groups was corrected using Welch’s correction. *P* < 0.05 was considered statistically significant.

## Results

### Post-thaw sperm quality analyses revealed a suboptimal cryopreservation protocol in the orangutan

**Fig. 1:**
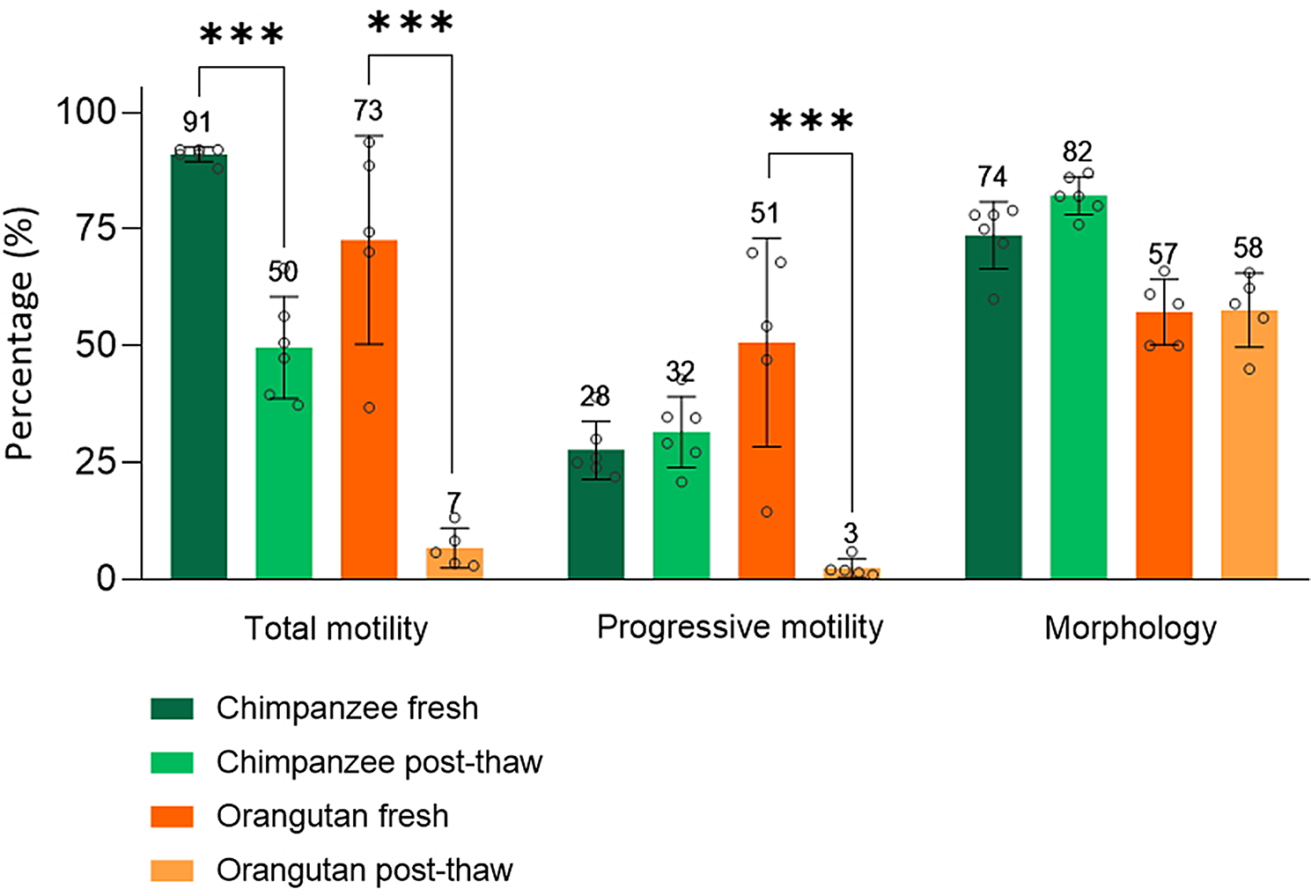
Post-thaw sperm quality evaluations of chimpanzee and orangutan ejaculate in response to the cryopreservation process. ***: *P* < 0.001

According to the post-thaw seminal analysis, cryopreserved orangutan sperm cells showed a significant decrease (*P* < 0.001) in total and progressive motility (-66% and − 48%, respectively) compared to fresh ejaculates (Fig. 1, Supplementary Table S1). Although a similar reduction phenomenon was observed in chimpanzee sperm, the degree of decrease in total motility was smaller (-41%) and not significant in progressive motility. Furthermore, similar to the results in chimpanzee, the morphology was not affected by the same cryopreservation protocol applied in chimpanzees (Fig. 1, Supplementary Table S1). Based on the data presented in Fig. 1, we considered the current cryopreservation protocol used in chimpanzees suboptimal for use in orangutans, as the total motility did not reach 40%. Consequently, we further investigated the factors that could cause the reduced cryopreservation resilience observed in orangutan ejaculates, with the objective of elucidating molecular or procedural limitations impeding post-thaw viability in orangutan.

### Pre- and post-cryopreservation sperm lipidomic profiles demonstrated species-specific alterations

**Fig. 2:**
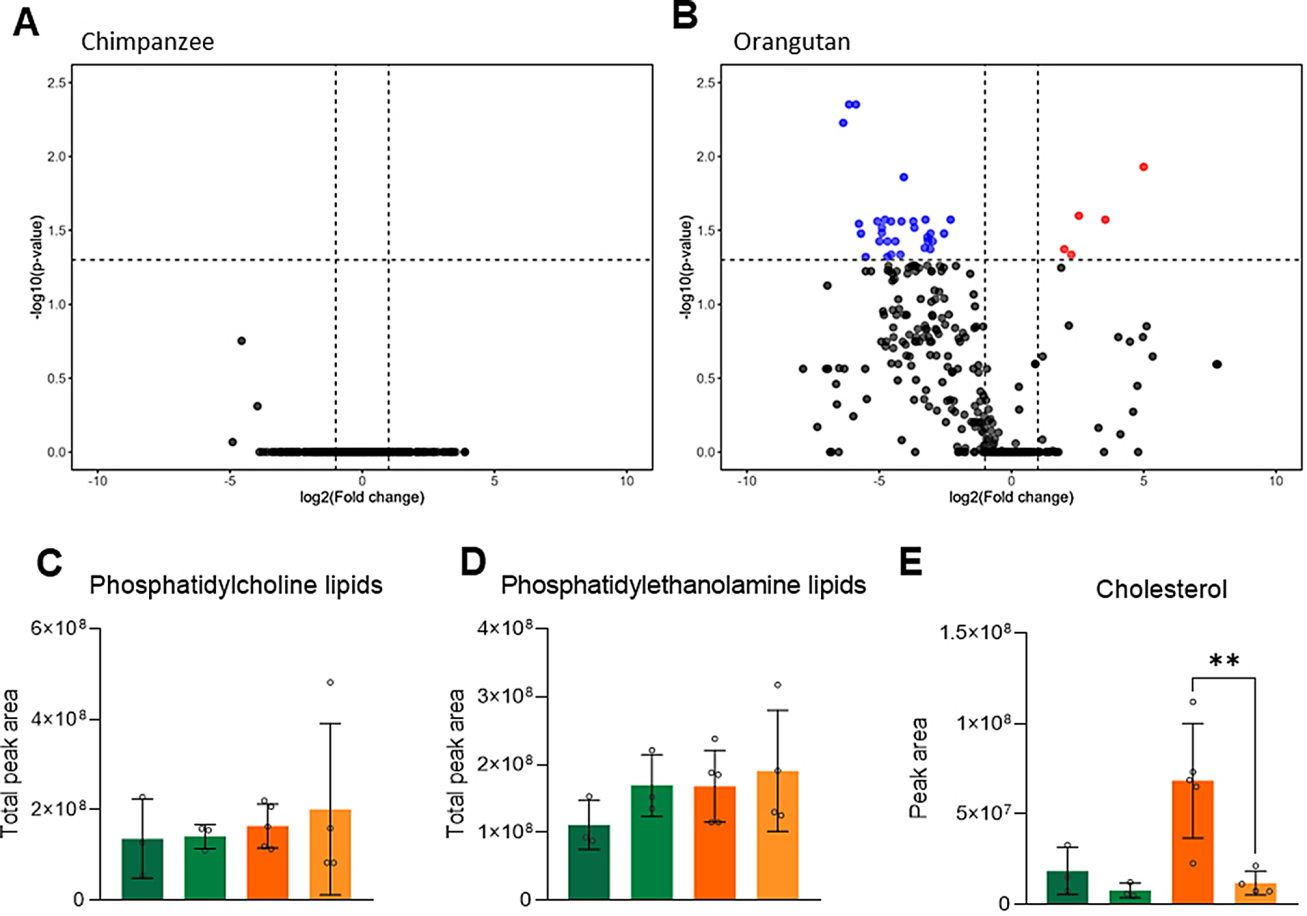
Lipidomic profiling of great apes’ sperm during the cryopreservation process. (**A**) Volcano plot of chimpanzee sperm lipid profile, fresh vs. post-thaw. (**B**) Volcano plot of orangutan sperm lipid profile, fresh vs. post-thaw. Individual data points (nodes) correspond to distinct lipid species; a decrease in relative abundance is denoted by blue, an increase by red, and no statistically significant change (|fold change| < 1.5) by grey. (**C**) Phosphatidylcholine lipid content did not show significant variation during the cryopreservation process across species. (**D**) Phosphatidylethanolamine lipid content did not show significant variation during the cryopreservation process across species. (**E**) Cholesterol content showed pronounced variation during the cryopreservation process across species. **: *P* < 0.01

Membrane lipid organization is crucial for the stability of sperm cells and significantly impacts sperm physiology [11]. To understand the possible causes behind orangutan ejaculation notwithstanding the cryopreservation process, we compared the complete lipidome of both chimpanzee and orangutan sperm through cryopreservation. According to the lipidomic profiling, the two species react distinctly to the same cryopreservation process (Fig. 2). As shown in the volcano plot, the chimpanzee lipid profile was not affected by the cryopreservation process (Fig. 2A); on the contrary, cryopreservation led to the decrease of 31 lipid species in orangutan sperm (Fig. 2B). Further analyses focused on the three main lipid classes of sperm membrane, phosphatidylcholine (PC), phosphatidylethanolamine (PE) and cholesterol, we observed that in both species, the phospholipid content was not affected by cryopreservation (Fig. 2C, D, Supplementary Table S2); however, cholesterol content in orangutan sperm decreased significantly (*P* < 0.01, Fig. 2E, Supplementary Table S2).

**Fig. 3:**
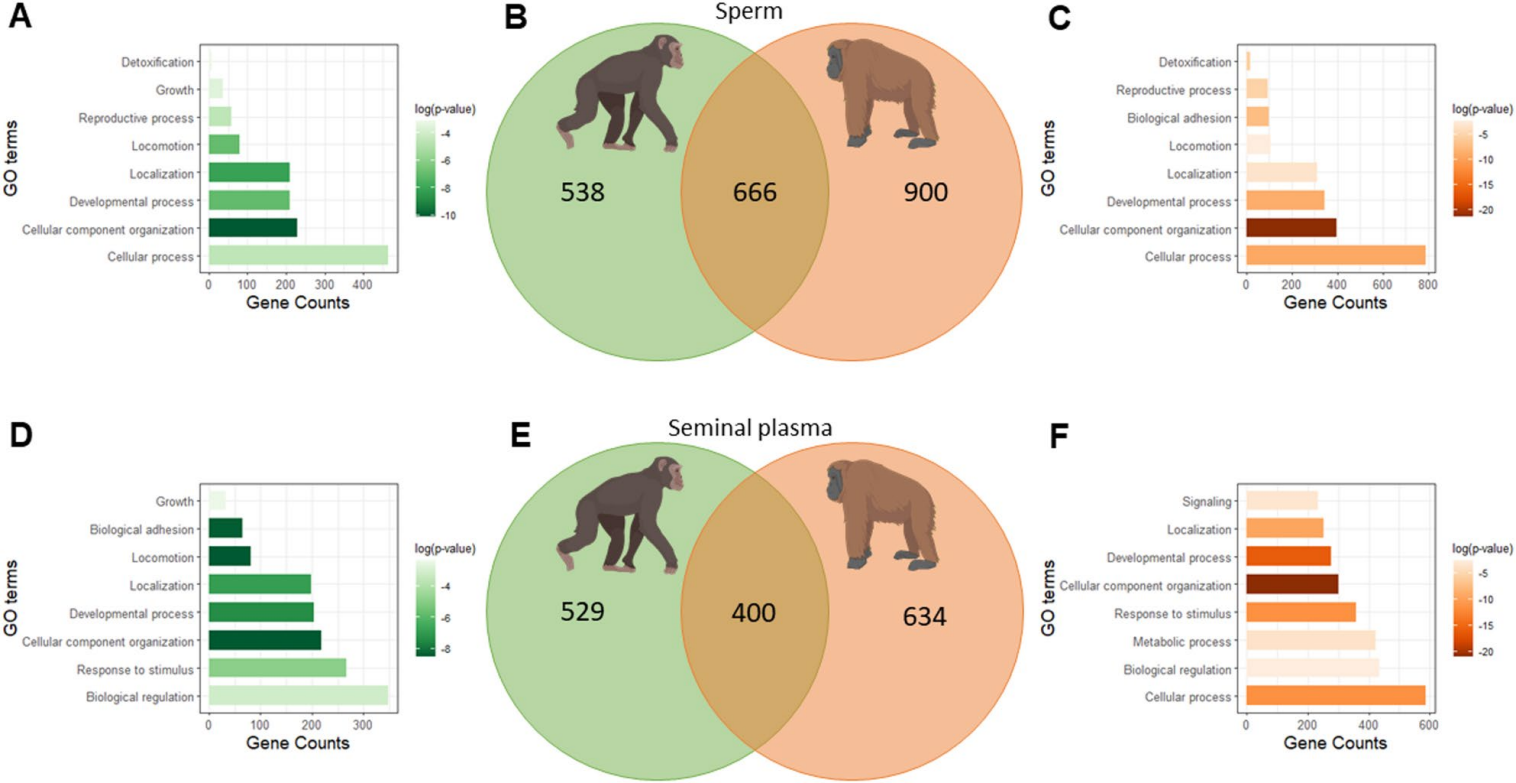
Comparative proteomic analysis of chimpanzee and orangutan ejaculates. (**B**) Common and species-specific sperm proteins and (**E**) common and species-specific seminal plasma proteins. Most common biological processes of (**A**) Chimpanzee sperm unique proteins, (**C**) Orangutan sperm unique proteins, (**D**) Chimpanzee seminal plasma unique proteins and (**F**) Orangutan seminal plasma unique proteins

### Proteomic analysis of great apes’ ejaculates demonstrated species-specific profiles

To understand the possible causes behind orangutan ejaculates notwithstanding the cryopreservation process, we revealed, for the first time, the complete proteome of spermatozoa and seminal plasma in both chimpanzee and orangutan. A total of 2133 proteins were detected in chimpanzee ejaculates, 1204 from spermatozoa, and 929 from seminal plasma (Fig. 3B, E, green circle). In orangutan ejaculates, 2600 proteins were identified, 1566 from sperm cells, and 1034 from seminal plasma (Fig. 3B, E, orange circle). In sperm cells, 666 proteins were commonly expressed in both species; in contrast 538 and 900 proteins were species-specifically present in either chimpanzee or orangutan sperm (Fig. 3B). In seminal plasma, 400 proteins were commonly expressed in both species, while 529, and 634 protein IDs were present specifically in either chimpanzee or orangutan seminal plasma (Fig. 3E). Most unique genes (chimpanzee-specific sperm, orangutan-specific sperm and seminal plasma) were strongly related to cellular component organization (Fig. 3A, C, D, F). On the contrary, chimpanzee seminal plasma unique genes were strongly related to biological adhesion and locomotion (Fig. 3D).

### Oxidative stress defense mechanisms evaluation revealed distinct antioxidant enzyme networks between chimpanzee and orangutan

**Fig. 4:**
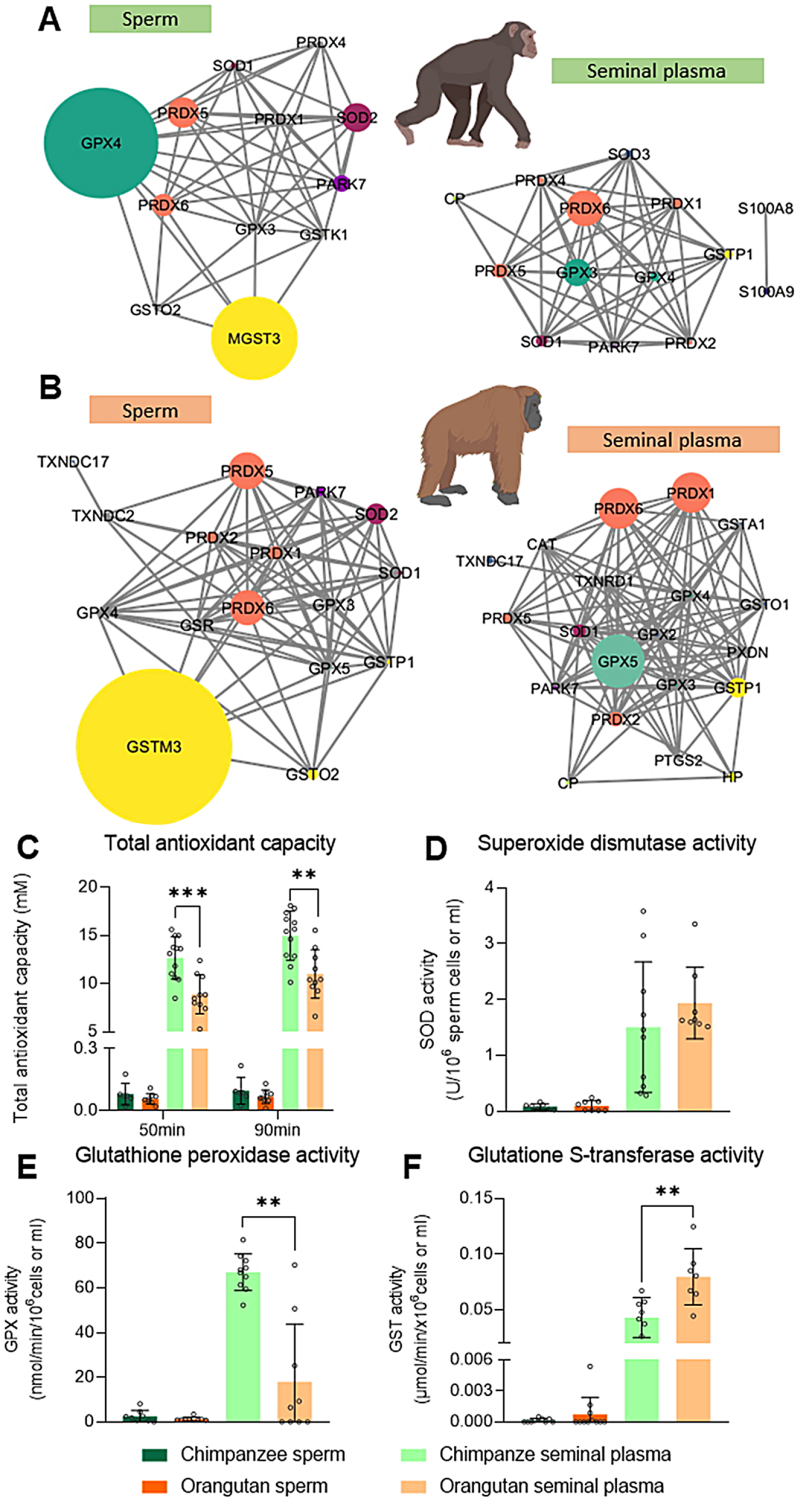
Proteomic profiling and evaluation of oxidative stress defense mechanisms of chimpanzee and orangutan ejaculates. (**A**) Quantitative visualization of the antioxidant network of chimpanzee ejaculate. (**B**) Quantitative visualization of the antioxidant network of orangutan ejaculate. Node sizes are proportional to emPAI values. (**C**) Total antioxidant capacity of chimpanzee and orangutan ejaculates. (**D**) Superoxide dismutase activity of chimpanzee and orangutan ejaculates. (**E**) Glutathione peroxidase activity of chimpanzee and orangutan ejaculates. (**F**) Glutathione transferase activity of chimpanzee and orangutan ejaculates. **: *P* < 0.01, ***: *P* < 0.001

During cryopreservation, reactive oxygen species are generated as metabolic byproducts, inducing oxidative stress that compromises cellular integrity [13]. To delineate interspecies variability in oxidative stress defense mechanisms, we characterized the antioxidant system and quantitatively assessed the TAC and the main antioxidant enzyme activity. Based on proteomic analysis, chimpanzee sperm were mainly composed of GPX 4 and microsomal GST3, whereas orangutan sperm contained high amounts of GST mu 3 and, in lesser amounts, peroxiredoxin 5 and 6 (Fig. 4A and B). In seminal plasma, chimpanzee’s contained high amounts of GPX3 and peroxiredoxin 6, whereas orangutan’s contained high amounts of GPX5, peroxiredoxin 1, and 6 (Fig. 4A and B). We noted the presence of an additional superoxide dismutase isoform, the extracellular form, in chimpanzee ejaculates, as well as the abundance of several glutathione transferase isoforms (GSTA1, GSTO1, GSTP1) in orangutan. Quantitative analysis of the TAC of the ejaculates revealed no significant interspecies variation in spermatozoa-associated antioxidant activity. However, seminal plasma exhibited marked interspecies divergence, with chimpanzee samples demonstrating significantly elevated TAC levels at two critical time intervals (50 min, duration of the cryopreservation protocol, *P* < 0.001; 90 min (as advised by the commercial kit, *P* < 0.01) (Fig. 4C, Supplementary Table S3). While no interspecies differences were detected in sperm cells, quantitative analysis of specific antioxidant enzymes from the seminal plasma yielded heterogeneous results. No significant differences in SOD activity were detected between the two species of interest (Fig. 4D, Supplementary Table S4). Regarding GPX activity, chimpanzee seminal plasma showed significantly higher activity (*P* < 0.01) than orangutan seminal plasma (Fig. 4E, Supplementary Table S4). GST activity patterns were, however, opposite to GPX, as orangutan seminal plasma showed significantly more activity (*P* < 0.01) than chimpanzee seminal plasma (Fig. 4F, Supplementary Table S4). Overall, seminal plasma in both species exhibits higher antioxidant activity than sperm cells (Fig. 4). These findings suggest species-specific adaptations in the redox buffering systems of seminal plasma.

### Sperm osmosis regulation evaluation demonstrated superior adaptive ability for chimpanzee sperm

**Fig. 5:**
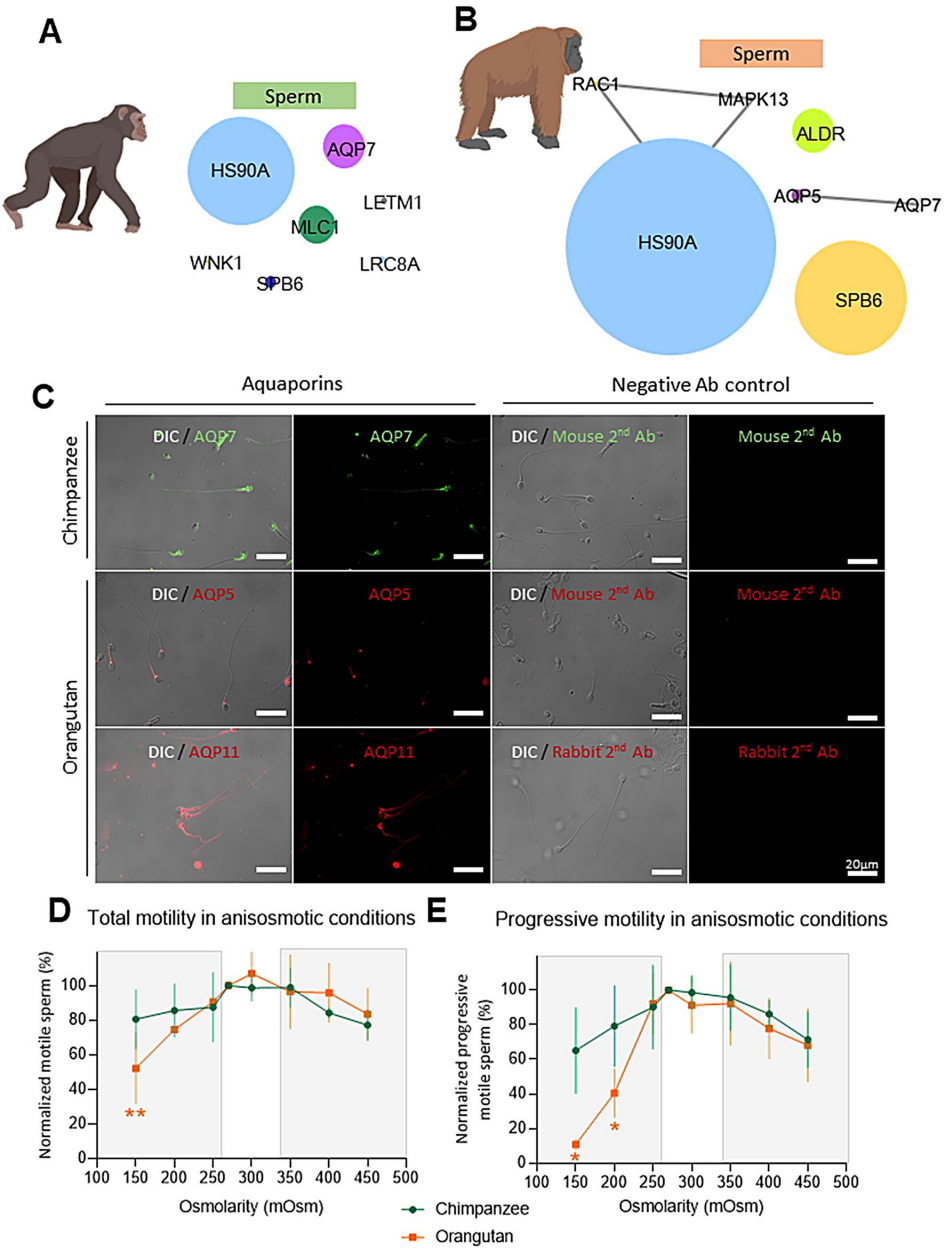
Proteomic profiling and evaluation of osmotic stress defense mechanisms of chimpanzee and orangutan sperm. (**A**) Quantitative visualization of osmotic stress response network in chimpanzee sperm. (**B**) Quantitative visualization of osmotic stress response network in orangutan sperm. Node sizes are proportional to emPAI values. (**C**) Cellular localization of aquaporins in great ape sperm cells. (**D**) Total motility in anisosmotic conditions, and (**E**) Progressive motility in anisosmotic conditions. *: *P* < 0.05, **: *P* < 0.01

Hyperosmotic stress occurs upon the addition of a cryoprotectant and the formation of ice crystals; on the contrary, hypoosmotic stress occurs during thawing when the physical state of water returns to liquid. Both stresses cause severe damage to the cells [13]. The protein candidates potentially involved in osmotic stress defenses in sperm were described in Fig. 5A and B. A noteworthy result was the presence of distinct aquaporin isoforms, a channel protein responsible for transporting water and other solutes, as chimpanzee sperm presented AQP7, and orangutan sperm contained AQP5 and AQP11. Through immunofluorescent staining, we uncovered in chimpanzee spermatozoa, AQP7 was uniformly distributed throughout the sperm (Fig. 5C). In orangutan spermatozoa, AQP5, a newly identified aquaporin in sperm cells, was localized exclusively on the midpiece, while AQP11 was detected in the post-acrosomal region and along the tail (Fig. 5C). To assess species-specific osmotic stress resilience in spermatozoa, sperm cells from each species were subjected to media of distinct osmolarities. Their functional resilience to osmotic changes was evaluated by measuring their motility. We observed that chimpanzee motility was not affected by either hypo- or hyperosmolar conditions (Fig. 5D and E). Whereas the orangutan was sensitive to and significantly affected by hypoosmolar conditions, as the total motility was significantly lower at 150mOsm (*P* < 0.01), with apparent decreased progressive motility from 200mOsm (*P* < 0.05, Fig. 5D and E).

### Modified thawing protocol exhibited significant improvement in post-thaw sperm motility

**Fig. 6:**
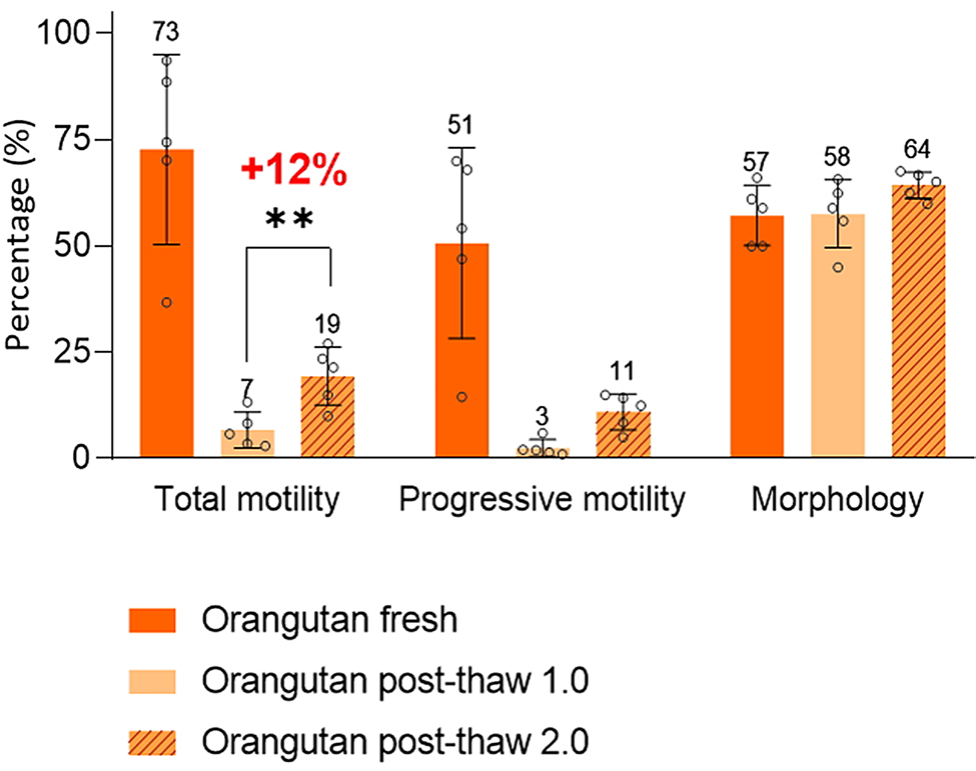
Comparative semen quality analysis in different thawing protocols. Post-thaw 1.0 consisted of the traditional abrupt osmotic transition thawing protocol used in chimpanzees. Post-thaw 2.0 consisted of a serial dilution of the thawed sperm. **: *P* < 0.01

Comparative lipidomic profiling of fresh and cryopreserved sperm revealed a significant depletion of membrane cholesterol content in orangutan spermatozoa during cryopreservation. Furthermore, proteomic profiling identified interspecies divergence in ejaculate protein composition. While both taxa exhibited distinct redox regulatory mechanisms, no definitive superiority in antioxidant efficacy was established. However, orangutan spermatozoa displayed attenuated osmotic stress defenses, rendering them more vulnerable to hypo-osmotic shock during glycerol removal upon post-thaw procedures. To minimize the above-mentioned effect, we applied a revised thawing protocol that employed serial dilution (post-thaw 2.0) rather than the traditional approach of abrupt osmotic transition (post-thaw 1.0). As shown in Fig. 6, though progressive motility remained statistically unchanged, this modification preserved better post-thaw motility (19%), a 12% improvement in total post-thaw motility in orangutan spermatozoa when compared to standard abrupt osmotic transition (post-thaw 1.0) protocols (*P* < 0.01; Fig. 6, Supplementary Table S5).

## Discussion

The Hominidae family, except for humans, faces imminent extinction risk, with current anthropogenic pressures disproportionately threatening their survival [2]. Among these, orangutans represent the most critically endangered lineage due to the synergistic effects of anthropogenic habitat fragmentation and intrinsically low fecundity rates, resulting in alarming population declines [4, 5]. While in situ conservation remains imperative to protect its natural habitat [4], it is becoming more evident that an *ex situ* conservation strategy must be established to safeguard the genetic diversity of this species. Current *ex situ* conservation efforts are limited by the fragmentation and small size of zoo populations. Moreover, live orangutan transport involves substantial logistical complexity and elevated risks of failure [23]. Consequently, the use of cryopreserved genetic material likely serves as a vital and feasible approach for *ex situ* conservation [24]. However, conventional cryopreservation protocols impose severe cellular trauma driven by ice crystal formation, membrane changes, oxidative damage, and osmotic shock [25]; moreover, the potential biological risk associated with egg yolk-containing (EYC) medium leads to the use of egg yolk-free (EYF) medium with similar post-thaw motility (EYC: 45% and EYF: 50% post-thaw total motility) [18]. In this study, we applied the same EYF sperm cryopreservation protocol used earlier in chimpanzees [18] to orangutan sperm, given their phylogenetic closeness. However, orangutan sperm showed unsatisfactory results, with total motility dropping 66% and progressive motility 48%, indicating the essential need to elucidate freeze-thawing-induced changes and assess the innate defense mechanisms against cryodamage.

**Fig. 7:**
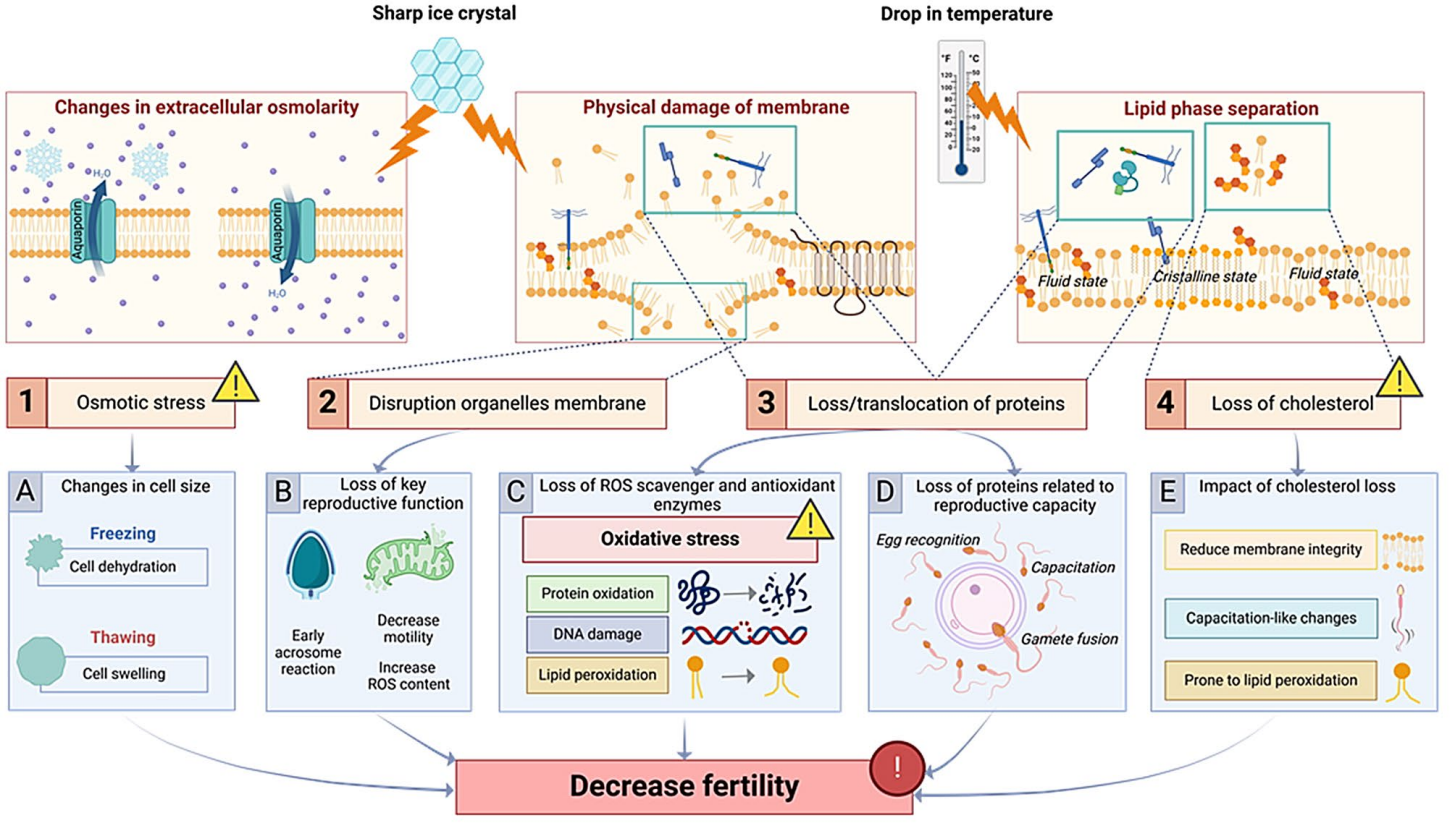
Proposed physiological mechanisms underlying cryodamages in orangutan sperm

Cryopreservation-induced changes in the lipid state are species-specific and can lead to a significant reduction in sperm fertilization capacity [13, 26]. In the present study, we showed significant differences in freezing-thawing lipid profiles between chimpanzee and orangutan sperm. Chimpanzee sperm lipid composition was stable throughout the freezing-thawing process; on the contrary, orangutan sperm was marked by a significant loss of lipids, specifically cholesterol content, after thawing, while the main phospholipids (phosphatidylcholine and phosphatidylethanolamine) remained unchanged, similar to an earlier study in buffalo [27]. Cholesterol is responsible for maintaining the membrane integrity, shaping the microenvironment of the surrounding membrane proteins, and reducing the permeability of membrane channels [28]. During the cryopreservation process, the changes in temperature lead to lipid phase separation and cause a considerable loss in cholesterol content (Fig. 7E), which in turn, leads to reduced membrane integrity, capacitation-like changes and makes other lipids more prone to lipid peroxidation [29] (Fig. 7E). It is likely that orangutan sperm is particularly sensitive to this cholesterol loss. Notably, some lipids were found to be in higher amounts in thawed sperm than in fresh sperm; this could partly be explained by freezing-thawing-induced structural changes in lipoproteins, which altered the density characteristics, or by cryopreservation-associated oxidative stress and its subsequent oxidation of unsaturated fatty acids [30].

During freezing and thawing processes, sperm cells are exposed to oxidative and osmotic stress (Fig. 7B-D) [10, 11]. Nevertheless, sperm and seminal plasma exhibit endogenous protective mechanisms, including enzymes and proteins, that counteract oxidative and osmotic stress. When comparing chimpanzee and orangutan, sperm cells and seminal plasma proteome showed 70% and 78% dissimilarities, respectively. These results are similar to other intra-family seminal plasma proteomes, where bull and ram presented 68% differences in their protein compositions, and ram and buck presented lower differences (52%) [31]. The high percentage of dissimilarities between chimpanzee and orangutan likely resulted from the early divergence of the orangutan from the rest of the Hominidae family (25 million years ago), making the orangutan a more distant chimpanzee’s relative [32]. During cryopreservation, excess oxidants are formed, and antioxidants are lost, resulting in severe oxidative stress [33] (Fig. 7C). Our proteomic data revealed that the antioxidant systems of the two species comprise different enzymes; however, in both cases, the system heavily relies on seminal plasma. This dependency is reasonable, as sperm cells lack cytoplasm and are transcriptionally inactive, making them poorly equipped to combat oxidative stress [34]. Although the total antioxidant capacity of chimpanzee seminal plasma was higher than that of orangutan, no specific antioxidant enzymes or activities showed a clear dominance. It is likely that the differences in antioxidant activity lie within other enzyme activities that have yet to be analyzed or non-enzymatic antioxidants [33]. When interpreting these proteomic differences, it is important to consider the limitations of the available reference databases. The chimpanzee and orangutan proteomes remain relatively incomplete; consequently, we used the human SwissProt proteome as a reference to enhance protein detection. This choice likely improved overall coverage but may have led to the underrepresentation of lineage-specific proteins, which should be considered when comparing species-specific antioxidant systems.

Apart from oxidative stress, the other main cause of low post-thaw quality results from osmotic stress [11]. During the freezing process, sperm cells are exposed to hyperosmolar condition due to the addition of cryoprotectant and the freezing of extracellular water, causing cell shrinkage (Fig. 7A). The reverse process takes place during thawing, lowering the extracellular osmolarity, causing a hypoosmolar environment and swelling of the cell (Fig. 7A) [11, 14], which have been reported as the most damaging factor upon cryopreservation process [10]. Chimpanzees presented volume-regulated ion channels (LRC8A) and water transport aquaporin (AQP7). LCR8A is responsible for sperm cell volume homeostasis [35]. AQP7, present on the whole chimpanzee sperm, has been related to facilitating rapid water movement across the membrane and volume regulation in mice [36]. Moreover, AQP7 is serve as a cryotolerance marker in boars [37], and it has been hypothesized to have the ability to transport glycerol [38]. In agreement with Agca and colleagues [39], we demonstrated in our osmotic tolerance curve that chimpanzee sperm exhibited high tolerance to the different anisosmotic conditions. On the other hand, the orangutan presented proteins related to hyperosmotic stress (RAC1) and water transport (AQP5 and 11). RAC1 is a protein that plays a role in the cellular response to hyperosmotic conditions [40, 41], suggesting that orangutan possess a protective mechanism against hyperosmolarity. This is evidenced by our osmotic tolerance evaluation, where orangutan presented a significant decrease in motility in hypoosmotic conditions but not under hyperosmotic conditions. Although the sperm proteome showed the presence of AQP5 in orangutan, and AQP5 has been associated with water homeostasis in the female reproductive tract and regulates the permeation of hydrogen peroxide in cancer cells [42], AQP5 has never been described in sperm cells. Thus, the exact functional relevance of AQP5 to volume regulation in orangutan sperm requires further investigation. It is worth noting that AQP5 is only present in the midpiece of orangutan sperm, where the mitochondria are located (principal producer of reactive oxygen species of the sperm cell [43]); it is likely that AQP5 we detected in orangutan sperm functions principally to transport hydrogen peroxide out of the mitochondria rather than water permeation. AQP11, presented in the orangutan sperm tail and post-acrosomal region, has been described as responsible for water efflux upon spermatogenesis in human and mice [44], as well as serving as a cryotolerance marker in bulls [45]. However, its exact function in mature sperm cells is unclear, as mice die at an early age of kidney failure in knockout studies [46]. Nevertheless, AQP11 has been hypothesized to have the capacity to transport hydrogen peroxide and serves as a modulator of redox homeostasis and signaling [22], based on its intracellular localization [44]. Taken together, our data suggest that the AQPs we detected in orangutan sperm may not be related to sperm volume regulation, but rather to the regulation of redox homeostasis. These results demonstrate that the chimpanzee is better equipped to withstand osmotic shock, possessing AQP7, a proven protector against osmotic changes, and exhibiting high tolerance to both hypo- and hyperosmolarity. On the contrary, orangutan sperm could defend against hyperosmotic shock but may not exhibit endogenous tools for volume regulation in mature sperm.

From the lipidomic analysis, we observed a significant loss in cholesterol, which potentially destabilizes the membrane. Earlier studies have demonstrated that using cholesterol-loaded cyclodextrin (CLC) to prevent cholesterol loss during cryopreservation improved outcomes [29, 47, 48]. We hypothesize that adding CLC to the extender will considerably improve the post-thaw quality in orangutan. On the other hand, in assessing osmotic defense mechanisms and sensitivity to osmotic changes, we demonstrated that orangutan sperm were sensitive to hypoosmolar conditions. Similar findings have been reported in human sperm [49]. This discovery led to modifications in our thawing procedure, as serial dilution of the thawed sperm has proven useful in minimizing hypoosmotic shock during the thawing process [50]. In our modified thawing protocol, we thaw orangutan sperm in a water bath at 37˚C for 10 s, followed by the slow addition (100 µl/min) of F-12 and processing at 25˚C. Satisfactory results were obtained, as the total motility was significantly higher in the optimized thawing protocol (12% improvement). These data underscore the critical role of osmoadaptive equilibration in cryopreservation outcomes for osmotically sensitive species, such as the orangutan.

## Conclusions

In conclusion, this study presents the first complete proteome and lipidome analysis of chimpanzee and orangutan ejaculates, providing valuable insights into the physiological changes and defense mechanisms associated with sperm cryopreservation. This knowledge enabled a science-based approach to improving cryopreservation protocols, moving away from trial-and-error methods. Specifically, we recommend incorporating cholesterol-loaded cyclodextrin to address the significant loss of cholesterol observed in orangutan sperm during cryopreservation. Additionally, we demonstrated that modifying the thawing protocol with a serial dilution effectively minimizes the hypoosmotic shock experienced by orangutan sperm.

## Electronic Supplementary Material

Below is the link to the electronic supplementary material.

Supplementary Material 1

## Data Availability

The proteomic dataset supporting the conclusions of this article is available in the ProteomeXchange Consortium via the PRIDE partner repository, [The unique persistent identifier is PXD073407 and hyperlink to dataset(s) in https:// format].
