# Supplementary material for: Cross-species multi-omic analyses of great ape ejaculates reveal novel strategies for enhancing sperm cryopreservation in Bornean orangutan (Pongo pygmaeus)
Source: Zoological Lett. 2026 Mar 6;12:7. doi: 10.1186/s40851-026-00262-x (PMC13081440; doi:10.1186/s40851-026-00262-x)
Supplement: Supplementary file 1 — Supplementary Material 1 [file 40851_2026_262_MOESM1_ESM.docx]

# **Cross-species multi-omic analyses of great ape ejaculates reveal novel strategies for enhancing sperm cryopreservation in Bornean orangutan (*Pongo pygmaeus)***

Laura Orama MÉAR^1,2^, Yu-Chia CHANG^3^, Jane-Fang YU^3^, Yun-Chen HSIEH^4^, Chia-Lin HSU^5^, Cheng-Chih HSU ^4,6^, Pei-Shiue TSAI^1,5,7,*^

^1^*Graduate Institute of Veterinary Medicine, National Taiwan University, No. 1, Sec. 4, Roosevelt Rd., 10617, Taipei, Taiwan*

^2^*Department of Reproduction Biology, Leibniz Institute for Zoo & Wildlife Research, No. 17, Alfred-Kowalke Rd., 10315, Berlin, Germany*

^3^*Conservation and Research Center, Taipei Zoo, No. 30, Sec 20, Xinguang Rd., Wenshan, Taipei, 11656, Taiwan.*

^4^*Department of Chemistry, National Taiwan University, No. 1, Sec. 4, Roosevelt Rd., 10617, Taipei, Taiwan*

^5^*Department of Veterinary Medicine, National Taiwan University, No. 1, Sec. 4, Roosevelt Rd., 10617, Taipei, Taiwan*

^6^*Leeuwenhoek Laboratories Co. Ltd., Taipei, Taiwan*

^7^*Research Center for Developmental Biology and Regenerative Medicine, National Taiwan University, No. 1, Sec. 4, Roosevelt Rd. 10617, Taipei, Taiwan*

^*^Corresponding author:

Pei-Shiue Tsai

E-mail: [psjasontsai@ntu.edu.tw](mailto:psjasontsai@ntu.edu.tw)

## **Supplementary Methods**


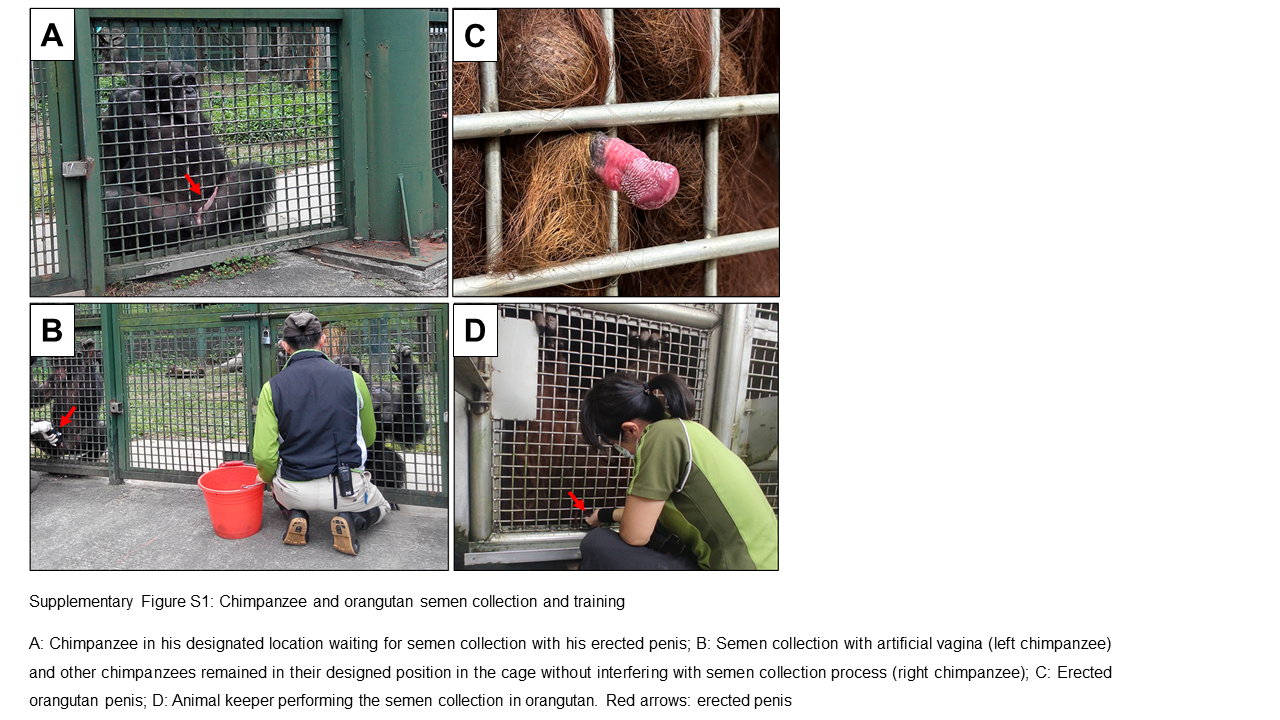


**Supplementary Figure S1: Chimpanzee and orangutan semen collection and training**

A: Chimpanzee in his designated location waiting for semen collection with his erected penis; B: Semen collection with artificial vagina (left chimpanzee) and other chimpanzees remained in their designated position in the cage without interfering with semen collection process (right chimpanzee); C: Erected orangutan penis; D: Animal keeper performing the semen collection in the orangutan. Red arrows: erected penis

**Sperm analysis**

For computer-assisted sperm analysis, the software and parameter settings were followed according to the recommendations of Hamilton Thorne Inc. Image capture was set to 60 frames per second, and a total of 45 frames were recorded per examination field. The default parameters and software were set up using human sperm parameters as a reference, with adjustments specifically for chimpanzee and orangutan use. At least 5 independent repeats were performed for each experimental condition; the mean value and standard deviation (SD) were calculated accordingly. Motility-related parameters, including total motility (%), and progressive motility (%, Hamilton system defined as VAP ≥25 µm/s, STR ≥30%), were measured and analyzed.

***Lipidomic analysis***

For lipidomic analysis, methyl tert-butyl ether (MTBE) was purchased from Thermo Fisher Scientific (HPLC grade; MA, US). Acetonitrile (ACN) and methanol (MeOH) were purchased from J.T. Baker (LC-MS grade, NJ, USA). Chemical standards, including 1-pentadecanoyl-2-oleoyl(d7)-sn-glycero-3-phosphocholine (15:0-18:1-d7-PC) and 1-pentadecanoyl-2-oleoyl(d7)-sn-glycero-[phosphorpho-rac-(1’-glycerol)] (15:0-18:1-d7-PG) were purchased from Avanti Polar Lipids (Alabaster, AL, USA). Ultrapure water was obtained using PureLab Classic (ELGA, UK).

The MTBE liquid-liquid extraction protocol, with laboratory modifications (1), was used to extract the sperm sample. An aliquot of 50 µL of the sperm sample was extracted by adding 600 μL of MTBE and 150 μL of MeOH. The sample was then spiked with 10 μL internal standards (IS) mixture containing 15:0-18:1-d7-PC (10 ppm), 15:0-18:1-d7-PG (10 ppm), and d6-Cholesterol (50 ppm). After that, the sample was vortexed (Vortex-Genie 2T, Scientific Industries) for 20 min at room temperature. Next, the sample was supplemented with 200 μL of water and centrifuged for 5 min at 13,684 × g to facilitate phase separation. The upper portion was transferred to another tube. The lower portion was extracted again by adding 100 μL of water, 100 μL of MeOH, and 300 μL of MTBE, and then vortexed for 10 min, followed by centrifugation. The upper portion was combined and dried in a vacuum concentrator (Vacufuge Plus Vacuum Concentrator, Eppendorf) for 3 hours, then stored at -80°C for further analysis. Before LC-MS analysis, the sample was reconstituted with 100 μL of reconstituting solution (ACN/IPA/H_2_O, v/v/v = 65/30/5) and sonicated for 3 min. A 20 μL of each sample was mixed to obtain a pooled quality control sample (QC).

Untargeted metabolomic analysis and cholesterol analysis were performed on an ultra-performance liquid chromatography system (Vanquish, Thermo Scientific) coupled with a high-resolution mass spectrometer (Orbitrap Elite, Thermo Scientific). A 5 μL sperm extract was separated using a Waters UPLC ACQUITY CSH C18 column (2.1 x 100 mm, 1.7 μm). The flow rate was set at 0.2 mL/min. The column temperature was set at 50°C. A binary gradient was used, with ACN/H_2_O (v/v = 4:6) as mobile phase A, and IPA/ACN (v/v = 9:1) as mobile phase B, each containing 0.1% formic acid (98% purity, Honeywell, NJ, USA). The composition was adjusted to the initial condition (25% B) for 30 minutes before the analysis began. The gradient was set as follows: 0-1 min, 25% B; 1-2 min, 25-70% B; 2-16 min, 70-99% B; 16-18.5 min, 99% B; 18.5-19 min, 99-25% B; and 19-21 min, 25% B. The composition was rebalanced during a subsequent wash gradient after each sample injection.

For full mass acquisition, a mass range of 200-1000 *m/z* was scanned under positive ion mode. The instrument was set as follows: (1) for untargeted metabolomic analysis, an Orbitrap Elite instrument coupled with a heated electrospray ionization (HESI) probe operated at a +3.5 kV spray voltage, 300°C heater temperature, 320°C capillary temperature, 40 arb sheath gas flow, and 12 arb auxiliary gas flow. (2) For cholesterol analysis, an Orbitrap Elite instrument coupled with an atmospheric pressure chemical ionization (APCI) probe operated at a +5 kV spray voltage, 275°C capillary temperature, 10 arb sheath gas flow, and 5 arb auxiliary gas flow, “APCI Corona On” was checked.

Data-dependent MS/MS (ddMS2) spectra were acquired on QC sample for the 10 most intense ions in a full mass scan by 2 *m/z* isolation width. Ions were fragmented via higher-energy collision dissociation (HCD) with a normalized collision energy (NCE) of 35. The Orbitrap mass analyzer was set at 60,000 (FWHM) resolving power for full mass acquisition and 30,000 for ddMS2 acquisition. Auto gain control (AGC) targets for full mass acquisition and ddMS2 acquisition were set to 3E6 and 5E4, respectively. A dynamic exclusion was enabled for 30 s in ddMS2 acquisition. Samples other than QC followed identical parameters without a ddMS2 analysis.

Samples of different groups were injected alternately for the LC-MS analysis, and QC samples were injected every 8 injections to allow QC correction during data processing. The volcano plots were constructed using the result table of Compound Discoverer, and MetaboAnalyst 5.0 online platform was utilized to perform principal component analysis (PCA). The criteria for significance were defined as a false discovery rate < 0.05 (Benjamini–Hochberg test) and fold change > 2. UPLC-MS/MS data were processed using Thermo Scientific Compound Discoverer v3.3 software for retention time alignment and peak area integration. Next, the integrated peak areas were normalized by employing the SERRF QC Correction node in the software to minimize the batch effect. For compound identification, Thermo Scientific Lipid Search software was used to identify lipid classes based on MS/MS information. Next, Phosphatidylcholines (PC) and Lysophosphatidylcholines (LPC) were annotated as PC group, and Phosphatidylethanolamines (PE) and Lysophosphatidylethanolamines (LPE) were annotated as PE group. The peak intensities for each feature in both groups were added up to yield total PC abundance and total PE abundance. GraphPad Prism (version 8.0.1; Boston, USA) was used to produce the related figures.

### ***Proteomic analysis***

After collection, liquefaction, and filtration, the diluted ejaculates were placed in an isotherm bag at 4˚C for transport to our laboratory. To separate seminal plasma from sperm cells, the ejaculates were centrifuged at 5000 × g, 4˚C for 10 minutes. Sperm cells were then washed several times with PBS. The protein concentration of samples (chimpanzee, orangutan seminal plasma, or sperm cells) was determined using Pierce™ BCA Protein Assay Kit (23225, Thermo Fisher Scientific, USA). To analyze the protein profiles of chimpanzee and orangutan ejaculates, a 10% gel was used to separate the proteins. After separation, the SDS-gel was stained with Coomassie blue (10.1% w/v Coomassie blue powder (F789-03, JT Baker, USA), 50% methanol, and 10% acetic acid dissolved in 500 ml DDW) for 1 hour at room temperature and de-stained with DDW overnight. The SDS-gel of each sample was cut into 4 pieces based on the molecular weight; a total of 16 pieces were obtained.

For the proteomic analysis, soluble protein samples were first denatured by reduction buffer (2mM DTE/ 8M urea) for 1 hour at 37°C, Lys-C (1 hour, 37°C), and trypsin (16 hours, 37°C) were subsequently used as the digestion enzymes, and the digested peptides were suspended in 0.1% formic acid and were desalted by zip tip. Liquid Chromatography with Tandem Mass Spectrophotometry (LC/MS/MS) was performed using an Orbitrap Fusion Mass Spectrometer (Thermo Fisher Scientific, USA). The mass spectrometry data files were analyzed using Mascot Daemon (version 2.6.0; Matrix Science, London, UK) and searched against the SwissProt *Pan troglodytes* and *Pongo abelii* protein databases (dated 2022/03). Only two missed cleavages were allowed, and a MASCOT score of 231 to identify proteins of interest was used (scores, E-value <0.05 were reported). Because these species-specific databases yielded very few confidently assigned spectra, reflecting their limited proteomic annotation, the data were therefore searched against the SwissProt *Homo sapiens* database (release 2022/04) to increase peptide-protein matching efficiency. These protein IDs were converted into gene IDs for future categorization and annotation of molecular function. The protein functions were analyzed through the DAVID (Database for Annotation, Visualization and Integrated Discovery) Functional Annotation Bioinformatics Microarray Analysis website (<https://david.ncifcrf.gov/>). Clusters related to cryodamage defense mechanisms were studied, and the protein-protein interactions from those clusters were analyzed using the STRING website (<https://string-db.org/>). R and Cytoscape were used to produce the related figures.

***Aquaporin distribution via indirect immunofluorescent staining***

For aquaporin 5 (mouse monoclonal, SC-514022) and aquaporin 7 (mouse monoclonal, SC-376407), the following protocol was applied. Sperm cells were fixed with 4 % paraformaldehyde (PFA) for 15 min at room temperature (RT). Permeabilization of fixed sperm was performed using 0.01 % Triton X-100 for 3 min at RT. The non-specific signal was reduced using 10 % BSA for 30 minutes at room temperature. Anti-aquaporin 5 and 7 at a dilution of 1/50 in 1 % BSA were used for 1h incubation at RT. Subsequently, Alexa Fluor 564 anti-mouse secondary antibody (dilution 1/150) was used for further incubation for 1h at RT. For aquaporin 11(aqp11-1101sp, FabGennic International, USA), the following protocol was applied. Sperm cells were fixed with 4% PFA for 15min at RT. Permeabilization of fixed sperm was performed using 0.1% Triton X-100 for 15 min at RT. The non-specific signal was minimized by 2 % BSA for 1h incubation at RT. Aquaporin 11 was labeled using rabbit anti-aquaporin 11 at a 1/150 dilution in 0.1 % BSA. The incubation was executed at RT for 3h. Subsequently, the secondary antibody Alexa fluor 564 anti-rabbit (1/150 dilution) was incubated for 1h at RT.

**Supplementary results**

**Supplementary Table S1**: Post-thaw sperm quality evaluations of chimpanzee and orangutan ejaculate in response to the cryopreservation process.

|  | Chimpanzee fresh | Chimpanzee post-thaw | Orangutan fresh | Orangutan post-thaw |
| --- | --- | --- | --- | --- |
| Total motility (%) | 91±1.6 | 50±10.9*** | 73±22.3 | 7±6.0*** |
| Progressive motility (%) | 28±6.1 | 32±7.5 | 51±22.4 | 3±3.5*** |
| Morphology (%) | 74±7.1 | 82±4.0 | 57±7.0 | 58±8.2 |

All the results are expressed as means ± standard deviation. ***: P<0.001, compared to fresh.

**Supplementary Table S2**: Content of different lipid classes in chimpanzee and orangutan sperm

|  | Chimpanzee fresh | Chimpanzee post-thaw | Orangutan fresh | Orangutan post-thaw |
| --- | --- | --- | --- | --- |
| PC | 1.3±0.87×10^8^ | 1.4±0.27×10^8^ | 1.6±0.49×10^8^ | 2.0±1.89×10^8^ |
| PE | 1.1±0.36×10^8^ | 1.6±0.45×10^8^ | 1.6±0.52×10^8^ | 1.9±0.89×10^8^ |
| Cholesterol | 1.1±0.12×10^7^ | 0.8±0.40×10^7^ | 6.8±3.1×10^7^ | 1.2±0.66×10^7^ ** |

All the results are expressed as means ± standard deviation. All results are expressed as total peak area. **: P<0.01, compared to orangutan fresh.

**Supplementary Table S3**: Total antioxidant capacity of chimpanzee and orangutan ejaculates

|  | Chimpanzee sperm | Orangutan sperm | Chimpanzee seminal plasma | Orangutan seminal plasma |
| --- | --- | --- | --- | --- |
| TAC (50min) | 0,0797± 0,0521 | 0,0563± 0,0252 | 12,7± 2,19 | 8,89± 2,04*** |
| TAC (90min) | 0,0957± 0,0647 | 0,0663± 0,0325 | 15,0± 2,55 | 11,0± 2,51** |

All the results are expressed as means ± standard deviation. All the results are expressed in mM. **: P<0.01, ***: P<0.001, compared to chimpanzee seminal plasma.

**Supplementary Table S4**: Antioxidant enzyme activity of chimpanzee and orangutan ejaculates

|  | Chimpanzee sperm | Orangutan sperm | Chimpanzee seminal plasma | Orangutan seminal plasma |
| --- | --- | --- | --- | --- |
| SOD (U/10^6^ cells or ml) | 0.1±0.05 | 0.1± 0.09 | 1.5±1.17 | 1.9±0.64 |
| GPX (mmol/min/10^6^ cells or ml) | 2.6± 2.66 | 1.2± 0.90 | 67.0±8.19 | 18.0±25.9** |
| GST (µmol/min/106 cells or ml) | 0.00014± 0.00019 | 0.00073± 0.0016 | 0.043± 0.018 | 0.080± 0.025** |

All the results are expressed as means ± standard deviation. SOD=Superoxide dismutase; GPX=Glutathione peroxidase; GST=Glutathione S-Transferase. **: P<0.01, compared to chimpanzee seminal plasma.

**Supplementary Table S5**: Comparative semen quality analysis in different thawing protocols.

|  | Orangutan fresh | Orangutan post-thaw 1.0 | Orangutan post-thaw 2.0 |
| --- | --- | --- | --- |
| Total motility (%) | 73±22.3 | 7±4.2 | 19±6.9** |
| Progressive motility (%) | 61±22.4 | 3±2.0 | 11±4.2 |
| Morphology (%) | 57±7.1 | 58±7.9 | 64±3.1 |

All the results are expressed as means ± standard deviation. Post-thaw 1.0 consisted of the traditional abrupt osmotic transition thawing protocol used in chimpanzees. Post-thaw 2.0 consisted of a serial dilution of the thawed sperm. **: P<0.01, compared to orangutan post-thaw 1.0.

**References:**

1. Wang WC, Huang CH, Chung HH, Chen PL, Hu FR, Yang CH, et al. Metabolomics facilitates differential diagnosis in common inherited retinal degenerations by exploring their profiles of serum metabolites. Nat Commun. 2024;15(1):3562.
